# Micro-computed tomography with contrast enhancement: An excellent technique for soft tissue examination in humans

**DOI:** 10.1371/journal.pone.0254264

**Published:** 2021-07-09

**Authors:** Jehoon O., Hyun-Jin Kwon, Tae-Hyeon Cho, Seung Hoon Woo, Yun-Hee Rhee, Hun-Mu Yang

**Affiliations:** 1 Department of Anatomy, Yonsei University College of Medicine, Seoul, Republic of Korea; 2 Beckman Laser Institute Korea, Dankook University College of Medicine, Cheonan, Republic of Korea; 3 Surgical Anatomy Education Centre at the Yonsei University College of Medicine, Seoul, Republic of Korea; Tarleton State University, UNITED STATES

## Abstract

Manual dissection and histologic examination are commonly used to investigate human structures, but there are limitations in the damage caused to delicate structures or the provision of limited information. Micro-computed tomography (microCT) enables a three-dimensional volume-rendered observation of the sample without destruction and deformation, but it can only visualize hard tissues in general. Therefore, contrast-enhancing agents are needed to help in visualizing soft tissue. This study aimed to introduce microCT with phosphotungstic acid preparation (PTA-microCT) by applying the method to different types of human tissue. Specimens from human cadavers were used to examine the orbicularis retaining ligament (ORL), nasolabial fold (NLF), and the calcaneal tunnel of the sole. Using PTA-microCT, relevant information of human structures was identified. In the ORL study, tiny and delicate ligamentous fibers were visualized in detail with multidirectional continuity. In the NLF study, complex structural formation consisting of various types of soft tissue were investigated comprehensively. In the calcaneal tunnel study, the space surrounded by diverse features and its inner vulnerable structures were examined without damage. Consequently, we successfully applied the PTA-microCT technique to the analysis of specific human soft tissue structures that are challenging to analyze by conventional methods.

## Introduction

For human anatomy research, manual dissection is commonly used to investigate human structures such as muscles and connective tissue, but delicate structures can be easily damaged during manipulation. Histologic examination in terms of staining procedures provides detailed morphological and compositional information at the micro-scale. Although manual dissection is a well-established approach that provides images at a high spatial resolution, there are several limitations: (i) it can lead to an unwilling dimensional change such as topographic distortion or destruction of the target structure, (ii) histological inspection is usually represented two-dimensionally, (iii) the scrupulous manipulation to reveal a target structure clearly is a laborious and time-consuming task [1,2]. Thus, there is a need for improved imaging techniques with a less invasive approach for morphological conservation of the tissue.

Conventional computed tomography (CT) is generally used in clinical practice but cannot differentiate small anatomical features [2], so micro-scale CT (microCT) was developed to investigate tiny anatomical structures. MicroCT enables a three-dimensional (3D) volume-rendered observation of minute features at a minimum voxel size of one micron. Researchers can avoid sample destruction and disfigurement during procedures by using this method. However, microCT can only visualize hard tissues and is constrained by the low level of intrinsic X-ray absorption of soft tissues. Therefore, several agents are needed to enhance a contrast of tissues for discriminating soft tissue structures.

Phosphotungstic acid (PTA), phosphomolybdic acid (PMA), iodine potassium iodide (Lugol’s solution), and osmium tetroxide are well-known contrast-enhancing agents, and there are several studies comparing these agents [3,4]. Despite its proficiency, osmium tetroxide is known for its high toxicity [5]. Although PTA and PMA are slower than Lugol’s staining method, they demonstrate better performance in staining collagenous structures [6]. A study has shown that PTA is slightly superior to PMA [7].

MicroCT with PTA preparation (PTA-microCT) seems to be the most conclusive and minimally invasive research method to visualize soft tissues by 3D imaging. Although this technique is often used for studying animals and human embryos, it has not yet been applied to various human studies. Our study aimed to review PTA-microCT techniques for various human tissues. Herein, we selected complex or delicate structures from human samples that have rarely been observed by conventional methods and we used them to validate the PTA-microCT technique. The detailed protocol, advantages, limitations, and applications of PTA-microCT are disclosed.

## Materials and methods

### Materials

Various types of human soft tissue were classified into three categories to evaluate the efficacy of PTA-microCT: (i) the orbicularis retaining ligament (ORL) represents a tiny and delicate structure that could be damaged in manual manipulation, (ii) the nasolabial fold (NLF) represents complex structures with varied histological composition that are difficult to comprehensively understand, (iii) the calcaneal tunnel of the sole represents an enclosed structure containing vulnerable features.

Specimens from Korean human cadavers were examined in this research. Some of them were already observed in our previous clinical studies. We comprehensively reinvestigate these samples for the methodological evaluation or recruited new samples. For the ORL study, twenty-two specimens from 11 non-embalmed cadaver heads were obtained (mean age 73.7 years). For the NLF study, twenty-four samples were obtained from 12 cadavers (mean age 80.3 years). For the calcaneal tunnel study, 21 embalmed cadavers and three non-embalmed cadavers (mean age 82.1 years) were used. The cadavers had no history of trauma or operative procedures in the related area of each study. All cadavers used in these studies were legally donated to the Surgical Anatomy Education Centre at Yonsei University College of Medicine. We obtained information, including age, sex, and cause of death, from the cadavers, and have received Approval of Exemption from the Institutional Review Board. All procedures were in accordance with the Declaration of Helsinki of the World Medical Association.

### PTA-microCT protocol

The general PTA-microCT protocol consisted of five steps, including 3D reconstruction for analyzing data.

#### Obtaining samples

Human tissues were harvested from fresh or embalmed cadavers, with no significant differences between them.

*ORL*. Obtained from the area inferior to a border of the lower eyelid and superior to 1 cm below the orbital rim, between the medial and lateral canthi, containing skin, subcutaneous tissue, muscle, fat, and periosteum.

*NFL*. Obtained from the area beside the ala nasi to the area above the oral commissure, containing 1 cm from the medial and lateral to the NLF.

*Calcaneal tunnel*. Obtained from the medial malleolar and plantar regions, containing the calcaneal tunnel and interfascicular septum (IFS). The skin, subcutaneous tissue, deep fascia, and superficial vascular structures were removed.

The sample size was restricted by the maximum scanning size of the microCT equipment (i.e., 7 cm^3^). Samples were fixed in 10% formalin immediately and preserved for approximately a week.

#### Preparation for staining

After fixation, samples were cut into thinner pieces to enhance the penetration of the staining solution. We only cut the ORL and NLF samples into three parts, because they included skin for investigating dermal attachment, but we excluded skin in the calcaneal tunnel samples, because the region of interest was deep to the dermis. Then, samples were dehydrated in serial ethanol solutions (30%, 50%, 70%) for one day each and retained in a fresh 70% ethanol solution until the next step.

#### PTA preparation

Preparation of the PTA staining was started several weeks before microCT scanning. An amount of 1% of PTA powder was diffused in 70% ethanol and mixed on a shaker at 55–60 rpm. Plastic containers with each sliced piece of sample were filled with PTA solution. Samples were soaked and mixed on a shaker during staining. The staining duration was predetermined, and was based on the dimension and character of the tissue (ORL and NLF: 1–2 cm; 5 to 7 days, Calcaneal tunnel: 5–6 cm; 2 weeks). PTA staining is known to last for approximately ten months [1], but it is suggested that scanning should be performed promptly to secure complete staining. After staining, samples were preserved in a fresh 70% ethanol solution until scanning.

#### MicroCT scanning

Samples were wrapped in parafilm to avoid desiccation during scanning, so as not to deform the shape. General scanning parameters for human soft tissue were a source voltage of 70 kV and source current of 114 μA under our laboratory conditions. Our device, the Skyscan^TM^ 1173 (Bruker, Kontich, Belgium), managed to scan down to 5 μm^3^ voxel size, but the extent can vary depending on the region of interest (ORL and NLF: 20 μm^3^, calcaneal tunnel: 30 μm^3^). The resolution of all images in this study was 2240 × 2240.

#### 3D reconstruction

After scanning, NRecon software (version 1.7.0.4, Bruker) was used for converting CT data into image files that could be handled on a computer. Ring Artifacts Reduction of 7 and Beam-hardening Correction of 40 were the parameters used for our samples. Converted serial sectional images were observed with 3D volume-rendering software. In this study, all samples were observed using CTvox (version 2.7, Bruker), a concise but powerful software for visualizing practical volume-rendered images. Once the converted dataset had been loaded, 3D analysis of internal structures was done by simply *Moving* and *Rotating* the plane of a *Clipping Box*, and by turning on the *Lighting* option to provide more realistic images.

## Results

Several musculoskeletal structures are difficult to examine by conventional methods, and we aimed to investigate those structures using PTA-microCT.

### Orbicularis retaining ligament

**Fig 1:**
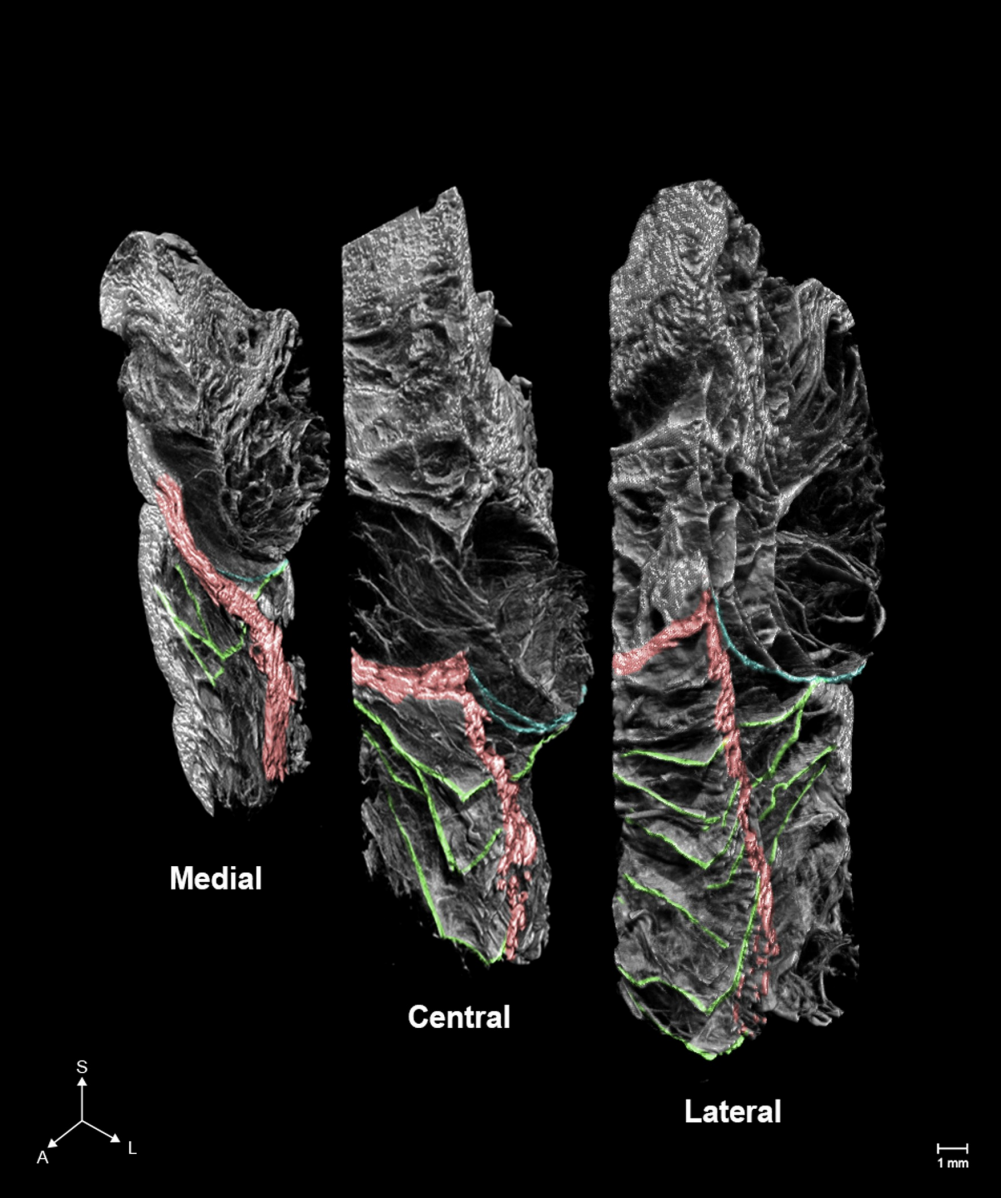
Micro-computed tomography (microCT) images of the orbicularis retaining ligament (ORL). The number, complexity, and ambiguity of the fibers comprising the ORL increased anteriorly and laterally. Green, blue, and red indicate the ORL, the orbital septum and, the section of the orbicularis oculi. S, superior; A, anterior; L, lateral.

The ORL is a structure around the orbit that sustains the eyelid and cheek skin. This ligament is a septal structure that begins from the periosteum, close to the orbital septum, and surrounds the lower orbital rim [8]. It is known for its muscular insertion to the orbicularis oculi and its cutaneous insertion to the malar region [9]. The ORL subdivides the midcheek subcutaneous space into the preseptal and prezygomatic-premaxillary spaces [10]. The deepening of the cutaneous groove can be induced by the sagging and shrinking of divided fat pads [11]. When the ligament has lost its tightness with aging, it can make the orbital fat drop and result in a preseptal area protrusion [9]. Since these morphological alterations can cause an adverse aesthetic effect on the aged face, manipulation of this ligament is essential for facial rejuvenation [10]. However, the ORL is too delicate to be damaged by manual dissection, while histological experiments can only visualize fragmentary images of the ligament. In this study, PTA-microCT could discern individual fibers with their multilayered structure, final attachment, and detailed 3D topography (Fig 1). Multidirectional microCT images confirmed that the ORL is a ligament between the periosteum and dermis which arborizes horizontally while crossing the orbicularis oculi muscle in an anteroinferior direction. The fibers increase from the medial to the lateral side, with its upper fibers showing a bolder appearance than the lower ones; the lowermost fibers reached the prezygomatic region. Comprehensively, the PTA-microCT image showed that the ORL was composed of multilayered plexiform plates with a broad range in a horizontal direction.

### Nasolabial fold

**Fig 2:**
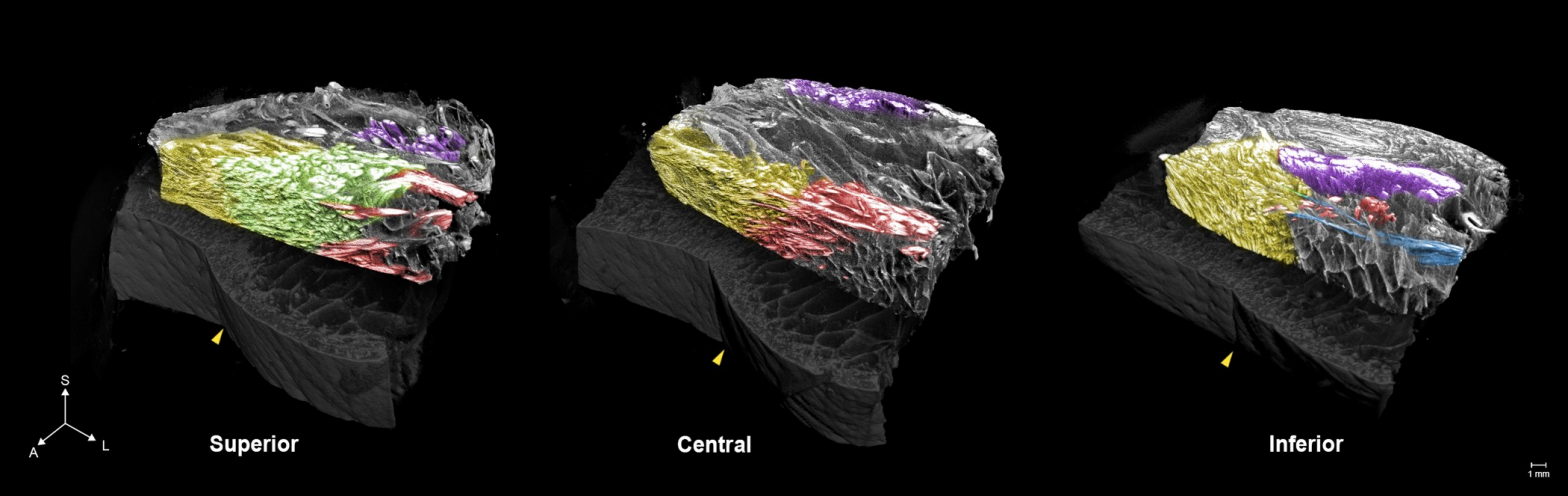
MicroCT images of the nasolabial fold (NLF). Arrangement and dermal attachment patterns of the muscles of the NLF are shown. Yellow, orbicularis oris; Green, levator labii superioris alaeque nasi; Red, zygomaticus minor; Blue, zygomaticus major; Purple, levator anguli oris; Yellow arrowhead, NLF.

The NLF is a fold that commences next to the nasal ala and ends at the oral commissure [12]. It had been known to be created by several anatomical factors such as repeated movement of muscles or an alteration of fat composition. Therefore, it is important to reveal which muscles attach to this fold and how the muscles function to understand the fold [13]. Moreover, the fold’s histological differences have been regarded as a prime factor representing the cutaneous groove; the medial dense fibromuscular complex and the lateral loose adipose-septum complex [14]. The deepening of the NLF caused by these factors leads to an aged appearance. Since the formation of the NLF is complex and constructed by delicate structures, 3D investigation of the fold is too subtle. Hence, we aimed to provide detailed 3D anatomy of the fold by PTA-microCT (Fig 2). In the alar region, the muscle complex of orbicularis oris (OOr) and levator labii superioris alaeque nasi were attached to the medial NLF, and its lateral margin corresponded to the fold. In the middle region, the OOr and the zygomaticus minor (ZMi) were attached to the medial NLF, and the intermuscular connective tissue divided the OOr-ZMi layer from the deep levator anguli oris muscle layer. In the angular region, the OOr and zygomaticus major were found, but neither of them were attached to the dermis, rather fibrous septa were attached both medially and laterally to the NLF. Furthermore, some dropping fibers of the ZMi were attached to the angular region.

### Calcaneal tunnel

**Fig 3:**
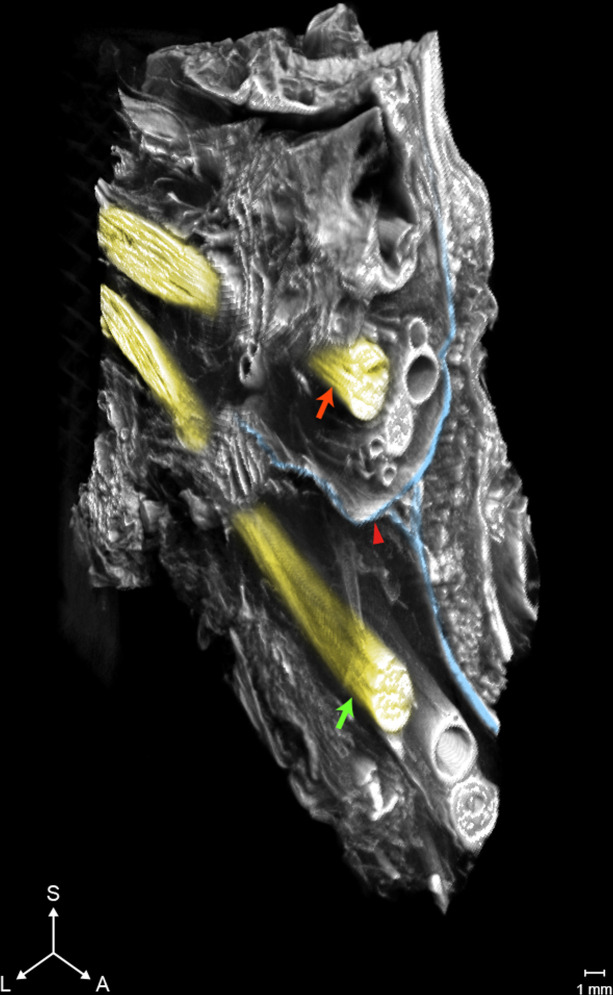
MicroCT images of the calcaneal tunnel. The interfascicular septum (IFS) separates the plantar nerves as the medial plantar nerve (MPN) and the lateral plantar nerve (LPN), which each innervate a space of the tunnel created by the IFS. Blue, IFS; Red arrow, MPN; Green arrow, LPN.

The calcaneal tunnel is a narrow space between the ankle bones and the flexor retinaculum, and plantar nerves pass through this tunnel [15]. Since plantar nerves innervate cutaneous sensation of the sole, abnormal condition or compression of the calcaneal tunnel can produce the pain in that area [16]. Previous studies have reported that the calcaneal tunnel is separated by a fascial septal structure and some authors have indicated that the medial and lateral branches of the plantar nerves (MPN and LPN) are localized by this septal structure, the IFS [17]. If so, surgeons should pay attention to this structure for pain reduction [18]. Since the tunnel and its internal structures are quite vulnerable and complex to observe by dissection, the relationship between the IFS and plantar nerves has remained obscure. Accordingly, we aimed to examine this space by PTA-microCT (Fig 3). The results confirmed that the IFS divided the calcaneal tunnel into superior and inferior regions, where the MPN and LPN were located within each compartment. The MPN and LPN had two or three small branches, but none of them penetrated the IFS.

## Discussion

PTA is known for its attractive interaction with collagen, and it has the potential for use in analyzing musculoskeletal tissues such as muscles, ligaments, and tendons [6].

Multidirectional observations using PTA-microCT provided detailed anatomy of the ORL that contradicts previous studies; the ORL is not composed of a single or double plates [19], but comprises a multilayered meshwork of fragile, continuous fibroelastic plates, and its lower fibers reach the prezygomatic region where it is known that the malar mound is produced by the zygomatic cutaneous ligament [20]. Therefore, we recommend that previous descriptions of midcheek cutaneous grooves needs to be reassessed because it is not induced by the demarcation of separate ligaments, but by complex factors related to the extensive ORL fibers with their adjacent subcutaneous and adipose tissue. The results of our study should be considered for midfacial rejuvenation and lower blepharoplasty.

In the case of the NLF, PTA-microCT revealed the comprehensive anatomy of the NLF; all muscle fibers attached medially to the fold, and it showed that the NLF is created not only by the pulling of the muscular attachment of the groove but also by the opposition between the lateral and medial aspects of the NLF [21]. Therefore, we suggest that the adipose tissue lateral to the fold, and the muscle traction medial to the fold should be considered to improve the fold. In addition, we found that neurovascular structures were located both laterally and medially to the fold, unlike the previous description [22]. These findings can help physicians who perform rejuvenation of the NLF using filler augmentation or botulinum toxin injection.

We investigated the calcaneal tunnel of the sole, the space surrounded by diverse features, and revealed its complicated anatomy without interfering with its inner delicate structures; the tunnel was separated by the IFS, and the medial and lateral plantar nerves were localized by each space [23]. Planter nerves are medicated to reduce pain of the foot and the findings of our study have raised an issue where the division between each branch, MPN and LPN, can undermine the efficiency of pain control [24]. Hence, we suggested that an analgesic injection to the plantar nerves for alleviating foot pain should be reconsidered.

This study confirmed that various types of human soft tissue structures can be examined by PTA-microCT. In the ORL study, tiny and delicate ligamentous structures was distinctly identified with three-dimensional continuity. In the NLF study, complex structures consisting of the septum, muscle, connective tissue, and related skin were investigated comprehensively. In the calcaneal tunnel study, the septum and neurovascular structures within the space surrounded by diverse features were examined non-invasively. PTA-microCT comprehensively complemented the limited macroscopic view of manual dissections, and the fragmented sectional views of histological examinations.

Several points were considered while processing the samples. The size and character of a specimen, and the duration of staining were principal concerns. Since the dimension of the specimen varied from the mm-scale to a maximum of 7 cm^3^, the scanning voxel size varied from under 10 to over 30 μm^3^. This intermediate-scale view, between macro and microscopic nanoscale view, is suitable for investigating anatomical structures of the human in detail [25]. However, if the dimension of a specimen is too large, the concomitant voxel size would lower the resolution of the image.

Regarding the type of tissue, ligamentous structures and muscles were stained and identified clearly, but the skin, which has higher density than the other tissues, needed to be stained for a longer duration for the PTA solution to penetrate. As for neurovascular structures, arteries and veins were easily identified for their notable, tubular shape. Small nerve twigs can be confused with other connective tissue or muscle fibers, so they should be carefully examined with anatomical understanding and using other verifications, such as histological experiments.

After several pilot studies, we found that the proper thickness of a specimen (including tissue of high density, such as skin) is 5–7 mm and the proper duration of staining is 5–7 days in most cases. In these conditions, the PTA solution penetrates the specimen at a rate of approximately 1 mm/day. If the thickness exceeds 7 mm, the processing time increases. When the duration of staining is insufficient compared to the volume of a specimen, the final image may include an empty hole in the central area of the specimen. This often occurs, especially at the skin level. Therefore, removing unnecessary skin can reduce the staining time and improve staining efficiency. Further study on the optimal duration for staining larger and more dense specimens could prove useful.

Other candidates for micro-level imaging exist, but all of them have limitations. Scanning electron microscopy and transmission electron microscopy nearly omit one spatial dimension. Optical projection tomography also provides 3D imaging, but it is limited by sample size. Micro-scale magnetic resonance imaging (microMRI) is also suitable for observing delicate soft tissues, but microMRI still provides insufficient resolution compared to microCT and is relatively expensive with limited accessibility [26]. Phase-contrast microCT has a great potential in not requiring contrast agents for soft tissues, but both synchrotron or laboratory-based phase-contrast microCT are expensive and have limited availability compared to a conventional microCT device [27].

There are a few limitations to the PTA-microCT study. First, this method can only be applied to sacrificed samples in the laboratory. Second, PTA needs more time to penetrate the tissue compared to an iodine-based agent, as it has a larger molecular size. Third, since images of microCT could only be visualized in grayscale, some findings need to be confirmed by histological experiments.

## Conclusions

This study aimed to introduce microCT with PTA preparation as an observation method to characterize human soft tissue. For this purpose, we chose several parts of the human body that are challenging to analyze by conventional methods and successfully verified the efficacy of the method in doing this.

Multidirectional observations using PTA-microCT demonstrated that the ORL is not just a single or double layered structure, but rather comprises a multilayered meshwork of thin, continuous fibroelastic plates. Further, we found that the lower fibers of the ORL reached the prezygomatic region, which was previously regarded as the zygomatic cutaneous ligament.

The NLF is a complex structure consisting of various types of tissue and PTA-microCT elucidated the comprehensive and detailed anatomy of the NLF. We found that all muscle fibers attached medially to the fold and showed that the NLF is created not only by the pulling of the muscular attachments of the groove but also by the opposition between the lateral and medial aspects of the NLF.

The calcaneal tunnel is a space surrounded by diverse features and we investigated its vulnerable inner structures non-invasively. The tunnel is separated by the IFS, and the medial and lateral plantar nerves were localized by each space.
